# Construction of a novel nomogram for predicting overall survival in patients with Siewert type II AEG based on LODDS: a study based on the seer database and external validation

**DOI:** 10.3389/fonc.2024.1396339

**Published:** 2024-06-07

**Authors:** Xiaohan Yu, Chenglin Bai, Yang Yu, Xianzhan Guo, Kang Wang, Huimin Yang, Xiaodan Luan

**Affiliations:** ^1^ General Surgery Department, Dandong Central Hospital, China Medical University, Dandong, Liaoning, China; ^2^ General Surgery Department, Dandong Central Hospital, Jinzhou Medical University, Dandong, Liaoning, China; ^3^ The First Ward of General Surgery, The Third Affiliated Hospital of Jinzhou Medical University, Jinzhou Medical University, Jinzhou, Liaoning, China; ^4^ General Surgery Department, Dandong First Hospital, Jinzhou Medical University, Dandong, Liaoning, China

**Keywords:** Siewert type II AEG, LODDS, SEER database, nomogram, prognosis

## Abstract

**Background:**

In recent years, the incidence of adenocarcinoma of the esophagogastric junction (AEG) has been rapidly increasing globally. Despite advances in the diagnosis and treatment of AEG, the overall prognosis for AEG patients remains concerning. Therefore, analyzing prognostic factors for AEG patients of Siewert type II and constructing a prognostic model for AEG patients is important.

**Methods:**

Data of primary Siewert type II AEG patients from the SEER database from 2004 to 2015 were obtained and randomly divided into training and internal validation cohort. Additionally, data of primary Siewert type II AEG patients from the China Medical University Dandong Central Hospital from 2012 to 2018 were collected for external validation. Each variable in the training set underwent univariate Cox analysis, and variables with statistical significance (p < 0.05) were added to the LASSO equation for feature selection. Multivariate Cox analysis was then conducted to determine the independent predictive factors. A nomogram for predicting overall survival (OS) was developed, and its performance was evaluated using ROC curves, calibration curves, and decision curves. NRI and IDI were calculated to assess the improvement of the new prediction model relative to TNM staging. Patients were stratified into high-risk and low-risk groups based on the risk scores from the nomogram.

**Results:**

Age, Differentiation grade, T stage, M stage, and LODDS (Log Odds of Positive Lymph Nodes)were independent prognostic factors for OS. The AUC values of the ROC curves for the nomogram in the training set, internal validation set, and external validation set were all greater than 0.7 and higher than those of TNM staging alone. Calibration curves indicated consistency between the predicted and actual outcomes. Decision curve analysis showed moderate net benefit. The NRI and IDI values of the nomogram were greater than 0 in the training, internal validation, and external validation sets. Risk stratification based on the nomogram’s risk score demonstrated significant differences in survival rates between the high-risk and low-risk groups.

**Conclusion:**

We developed and validated a nomogram for predicting overall survival (OS) in patients with Siewert type II AEG, which assists clinicians in accurately predicting mortality risk and recommending personalized treatment strategies.

## Introduction

Adenocarcinoma of the esophagogastric junction (AEG) refers to adenocarcinoma whose tumor center is within 50 mm above and below the esophagogastric junction (EGJ), i.e., distal esophagogastric junction and proximal gastric junction and invades the EGJ. The specific anatomical location of adenocarcinoma causes its biological behavior to be different from that of esophageal and gastric cancers (1, 2). Currently, there are two main types of AEG classification: Nishi classification (3) and Siewert classification (4). At present, the Siewert type proposed by German scholars Siewert and Stein is widely used in the international community, which classifies AEG into three types according to the location of the tumor center: Type I: the tumor center is located in the range of 10–50mm above the EGJ and the tumor infringes on the EGJ; Type II: the tumor center is located in the range of 10mm above and 20mm below the EGJ and the tumor infringes on the EGJ; Type III: the tumor center is located in the range of 10mm above and 20mm below the EGJ and the tumor infringes on the EGJ. In recent years, due to the increasing trend of gastroesoph60al reflux disease (GERD), obesity, smoking and Helicobacter pylori infection, the incidence of adenocarcinoma of the esophagogastric junction (AEG) has increased rapidly worldwide (5–7). Despite continuous advances regarding the diagnosis and treatment of AEG, and radical surgical resection remains the predominant treatment, the survival rate of patients after AEG is still lower than ideal, with a five-year postoperative survival rate of only 25–47 percent (8, 9). The introduction of personalized treatment has brought attention to the prognostic factors affecting cancer patients. Therefore, it is of great significance to analyze the prognostic risk factors of Siewert type II AEG patients and construct a survival prediction model to guide precise and individualized treatment.

Currently, the TNM staging system is widely used to guide treatment regimens and assess patient prognosis (10). However, relying on the TNM staging system alone has serious drawbacks, as it contains only a limited number of tumor-related variables and has limitations in specificity, making it unsuitable for personalized analysis. The nomogram is a reliable statistical model that visualizes the interrelationships between variables in a predictive model by integrating multiple predictors on the same plane based on multifactorial regression analysis. A score is assigned based on the level of risk associated with each variable, and the sum of all scores ultimately represents the expected chance of survival (11). Studies have shown that the nomogram has better predictive performance than TNM staging, and various he nomogram has been used to predict the prognosis of different types of cancer, Lv et al. constructed a new nomogram for predicting the prognosis of patients with obstructive colorectal cancer (12). Wu et al. developed and validated a prognostic nomogram to help clinicians assess the overall survival of patients with low-grade endometrial mesenchymal sarcoma (13). Huan et al. developed a nomogram based on the seer database to predict overall survival in young breast cancer patients (14). However, few studies have focused on the prognosis of patients with Siewert type II AEG.

+The Surveillance, Epidemiology, and End Results (SEER) database, established by the National Cancer Institute, is one of the world’s most recognized oncology databases. It contains clinical and survival data from 18 population-based cancer registries, accounting for approximately 28% of U.S. cancer patients, and is now widely used in clinical research, providing an effective tool for tumor epidemiology studies (15).

This study aimed to extract clinical information from the SEER database and apply it to the analysis of prognostic risk factors for Siewert type II AEG. The nomogram was constructed to predict OS in patients with Siewert type II AEG and evaluated by internal and external validation.

## Information and methodology

### Data collection

Our study is based on the SEER database, using the SEER*stat software (version 8.4.2) for data retrieval (https://seer.cancer.gov/data-software/), which obtained data on patients with primary Siewert type II AEG from 2004 to 2015. Although the SEER database does not provide detailed information on the Siewert-type classification of AEG, the data were analyzed by the “Primary Site” code of 16.0 (cardia) and the “CS site-specific factor 25 “ coded as 982 (esophagus, gastroesophageal junction) allowed us to obtain a Siewert type II AEG (16). Patient inclusion criteria were as follows: (1) Unique primary malignancy. (2) patients with histologically confirmed AEG (according to ICD-O-3, 8140–8147, 8160–8162, 8180–8221, 8245, 8250–8507, 8514–8551, 8571–8574, 8576, 8940–8941), (3) age >18 years old, and (4) patients underwent radical surgery. The exclusion criteria for patients were as follows:(1) TNM stage was unknown or T stage was TX/T0/Tis. (2) The number of NX or cleared lymph nodes (ELN) and the number of positive lymph nodes (PLN) were unknown. (3) Tumor size unknown. (4) Tumor grade unknown. (5) Loss to follow-up or follow-up time less than 1 month. (6) Cause of death unknown. Ethical review was waived by the local ethics committee as the SEER database is publicly accessible and the data were de-identified.

**Figure 1:**
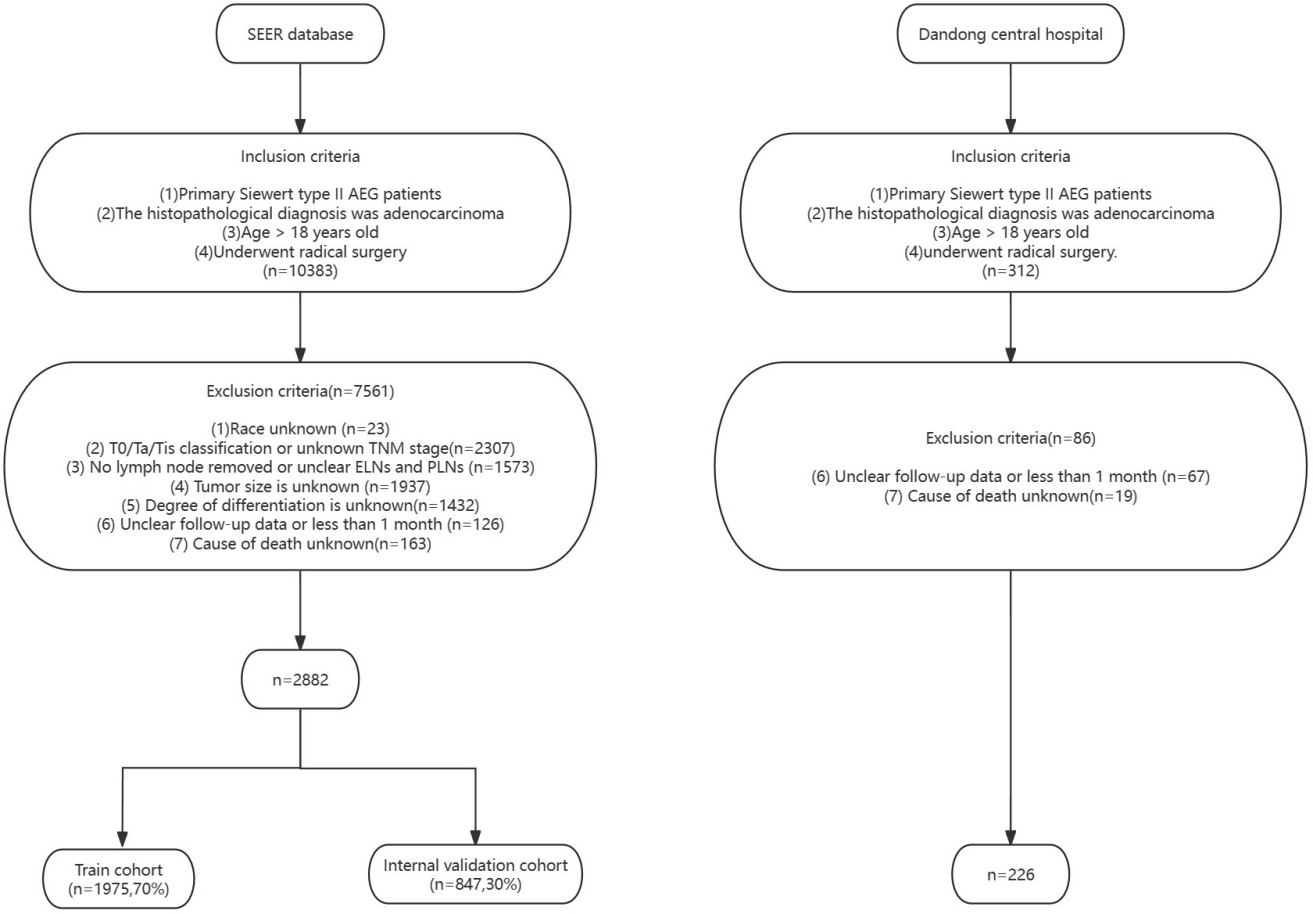
Flowchart of SEER database and external validation data selection process.

External validation data were obtained from the Dandong Central Hospital affiliated with China Medical University, and data related to the consultation information and baseline information of Siewert type II AEG patients diagnosed from January 2012 to December 2017 were collected using consecutive electronic cases. Survival follow-up of the patients was conducted through outpatient visits with review or telephone contact, with a final follow-up date of December 2023. Patient inclusion criteria were as follows: (1) Unique primary malignant tumor. (2) Histologically confirmed. (3) Age >18 years, and (4) patients underwent radical surgery. Patient exclusion criteria were as follows: (1) Duration of follow-up unknown or less than 1 month. (2) The cause of death was unknown. This study was approved by the Ethics Committee of Dandong Central Hospital, and the patient medical records were kept strictly anonymous, confidential and by the ethical standards of the Declaration of the World Medical Association of Helsinki. The specific screening process is shown in **Figure 1**.

### Collection indicators

**Figure 2:**
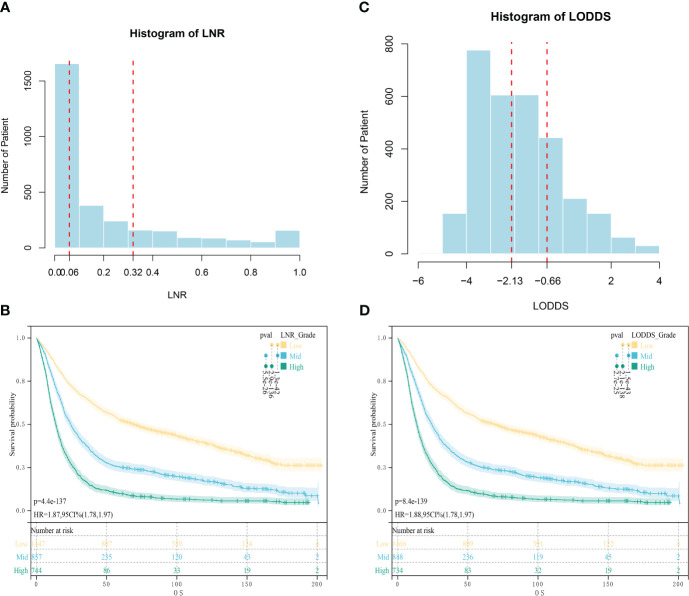
Optimal cut-off values obtained by X-tile calculation. **(A)** Optimal Cut-off Value for LNR; **(B)** Survival analysis of patients in different LNR groups; **(C)** Optimal Cut-off Value for LODDS; **(D)** Survival analysis of patients in different LODDS groups.

The code data extracted from the SEER database were translated according to the tumor code nomenclature manual provided by the U.S. National Cancer Institute and the International Classification of Diseases and Tumors Morphological Codes, 3rd edition (International Classification of Disease for Oncology, 3rd edition, ICD-O-3). Data from the external validation cohort were obtained through the hospital’s medical record system. Variables collected for this study were categorized using four main categories: patient-related variables, disease-related variables, treatment-related variables, and follow-up data. (1) Patient-related variables: age, gender. (2) Disease-related variables: histological type, tumor grade, 7th edition AJCC clinical staging (TNM), tumor size, ELN, PLN, lymph node ratio (LNR), and LODDS. (3) Treatment-related variables: neoadjuvant chemotherapy/adjuvant chemotherapy, neoadjuvant radiotherapy/adjuvant radiotherapy. (4) Follow-up information: survival status, cause of death, and survival time. The specific stratification was as follows: age was classified as ≤60 years old and >60 years old; histological type was classified as adenocarcinoma, Stamped Rectal Cell Carcinoma (SRC), and others; tumor grading was classified as: highly differentiated, moderately differentiated, poorly differentiated, and undifferentiated; tumor size was classified as s1 cm, 1–3 cm, 3–5 cm, and >5 cm; and T stage, N stage, and M stage were categorized in accordance with the 7th edition of the AJCC TNM Tumor Staging Manual; X-tile was used to calculate the optimal threshold for LNR and LODDS (17),LNR staging was categorized according to the cutoff values of 0.06 and 0.32 obtained by X-tile as LNR1<0.06, LNR2 as 0.06~0.32, and LNR3 as >0.33; LODDS staging was categorized according to the cutoff values of -2.13 and -0.66 obtained by X-tile as LODDS1<-2.13, -2.12 to -0.66 for LODDS2,and >-0.66 for LODDS3 (**Figure 2**); chemotherapy and radiotherapy were classified according to whether or not they received the treatment. LNR is the PLN divided by the ELN counts. The LODDS formula is log ([PLN + 0.05]/[ELN - PLN + 0.05]), and in both the numerator and the denominator 0.05 is added to avoid irrational numbers.

### Construction and validation of the nomogram

Patient data collected in the SEER database were randomly assigned in a 7:3 ratio to a training cohort (for construction of the nomogram) and an internal validation cohort (for validation of models constructed by the training cohort). Univariate Cox analyses were performed for each variable in the training cohort, and statistically significant variables (p < 0.05) were included in the LASSO equation for feature selection. In addition, multivariate Cox analyses were performed to identify independent predictors. A two-sided p < 0.05 was considered statistically significant. The primary endpoint of this study was overall survival (OS), defined as the duration from cancer diagnosis to death from any cause, based on multivariate Cox regression analysis, and the nomogram were constructed using the RMS and survival packages of the R software to predict the OS of the patients. The reliability of the model was verified by internal and external validation cohorts. The reliability of the model was verified by internal and external validation cohorts, and the “pROC” software package was used to plot the receiver operating characteristic curve (ROC) of the nomogram, and the area under the receiver operating characteristic curve (AUC) was used to predict the OS of patients. Calibration curves were constructed using the “rmda” software package to calculate the difference between the expected risk and the actual risk, and the closer the curves were, the more accurate the prediction was. Decision curve analysis (DCA) was constructed using the “rmda” software package to measure the net benefit at different threshold probabilities to assess the clinical effectiveness and usefulness of the model. In addition, to compare the constructed line graph model with the traditional TNM staging system, the Net reclassification index (NRI) and Integrated discrimination improvement (IDI) were calculated in the present study, and the NRI and IDI could be used to evaluate the new predictive model. The NRI and IDI can be used to evaluate the improvement of new prediction models relative to existing models (18, 19). Patients were categorized into high-risk and low-risk groups based on the median risk score. Survival curves for the high-risk and low-risk groups were plotted using Kaplan-Meier analysis, and the difference between the survival curves was assessed using the log-rank test.

### Statistical analysis

Descriptive statistics were used to summarize baseline characteristics. Categorical variables were expressed as frequencies and percentages (n, (%)), and differences between groups were analyzed using the chi-square test or Fisher’s exact test. SPSS (27.0) and R (4.2.1) software were used for statistical analysis and plotting, and two-sided P<0.05 was statistically significant.

## Result

### Clinicopathologic features of the patient

Data from 2822 patients with Siewert type II AEG were obtained from the SEER database and randomized in a 7:3 ratio to a training cohort (1975 patients) and an internal validation cohort (847 patients). In the external validation cohort, data were collected from 226 patients with Siewert type II AEG. In all three cohorts, the majority of patients were older than 60 years (62% vs. 60% vs. 61%), predominantly male patients (81% vs. 81% vs. 76%), and more than half of the tumors were poorly differentiated in terms of tumor grade (57% vs. 59% vs. 55%). The overall grouping of the training group, internal validation group and external validation group was consistent with the simple randomized grouping, and the difference between groups was not statistically significant. Detailed information is shown in **Table 1**.

**Table 1:** Baseline characteristics of patients in the training group, internal validation group, and external validation group.

Variables	Training Group (n = 1975)	Internal Validation Group (n = 847)	External Validation Group (n=226)	*P* *Value*	*FDR*
**Age, n (%)**				0.67	1.00
≤60	752 (38)	337 (40)	89(39)		
>60	1223 (62)	510 (60)	137(61)		
**Sex, n (%)**				0.21	1.00
Female	378 (19)	160 (19)	54 (24)		
Male	1597 (81)	687 (81)	172(76)		
**Histology, n (%)**				0.17	1.00
Adenocarcinoma	1460 (74)	630 (74)	167(74)		
SRC	209 (11)	104 (12)	32(14)		
Others	306 (15)	113 (13)	27(12)		
**Grade, n (%)**				0.71	1.00
Well differentiated	107 (5)	41 (5)	8(4)		
Moderately differentiated	689 (35)	288 (34)	85(38)		
Poorly differentiated	1131 (57)	500 (59)	125(55)		
Undifferentiated	48 (2)	18 (2)	8(3)		
**T, n (%)**				0.54	1.00
T1	369 (19)	153 (18)	44(20)		
T2	1041 (53)	434 (51)	118(52)		
T3	475 (24)	205 (24)	54(24)		
T4	90 (5)	55 (6)	10(4)		
**N, n (%)**				0.27	1.00
N0	668 (34)	275 (32)	72(32)		
N1	948 (48)	410 (48)	114(50)		
N2	265 (13)	135 (16)	33(15)		
N3	94 (5)	27 (3)	7(3)		
**M, n (%)**				0.82	1.00
M0	1829 (93)	790 (93)	210(93)		
M1	146 (7)	57 (7)	16(7)		
**Tumor size, n (%)**				0.44	1.00
≤1 cm	135 (7)	60 (7)	13(6)		
1–3 cm	620 (31)	262 (31)	82(36)		
3–5 cm	631 (32)	257 (30)	58(26)		
> 5 cm	589 (30)	268 (32)	73(32)		
**LNR, n (%)**				0.88	1.00
1	944(48)	395(47)	108(48)		
2	560(28)	234(27)	63(28)		
3	471(24)	218(26)	55(24)		
**LODDS, n (%)**				0.87	1.00
1	955 (48)	403 (48)	108(48)		
2	554 (28)	229 (27)	63(28)		
3	466 (24)	215 (25)	55(24)		
**Radiation, n (%)**				0.13	1.00
None/Unknown	998 (51)	393 (46)	110(49)		
Yes	977 (49)	454 (54)	116(51)		
**Chemotherapy, n (%)**				0.82	1.00
None/Unknown	658 (33)	294 (35)	77(34)		
Yes	1309 (67)	551 (65)	149(66)		

### Independent prognostic factors

**Figure 3:**
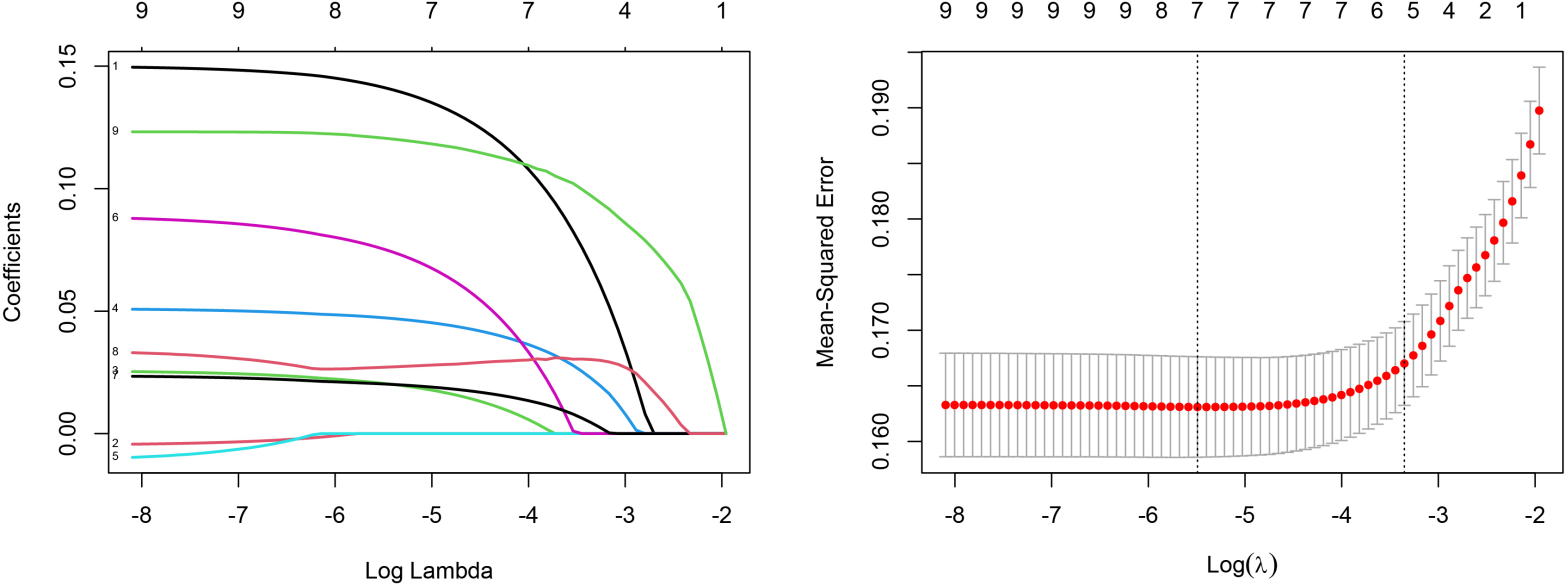
LASSO coefficients of 9 features and tuning parameter selection for LASSO model.

Univariate Cox regression analysis showed that age, histological type, tumor grade, T-stage, N-stage, M-stage, tumor size, LNR, and LODDS score were associated with overall survival (OS) (**Table 2**). To avoid overfitting, LASSO regression was performed using these nine variables, and the coefficient of histologic type, N stage, was zero, so it was excluded from the model (**Figure 3**). In multifactorial Cox analysis, the older the age (HR [95%CI] = 1.563[1.402–1.744], P<0.001), the lower the degree of differentiation (Grade III, HR [95%CI] = 1.363[1.051–1.767], P=0.019), and the higher the T-staging (T2, HR [95%CI] = 1.344 [1.125–1.605], P<0.001; T3, HR[95%CI] = 1.518 [1.249–1.845], P<0.001; T4, HR[95%CI] = 1.897 [1.422–2.531], P<0.001), the distant metastases (HR[95% CI] = 1.658 (1.379–1.992), P<0.001), and higher LODDS stage (LODDS2, HR[95%CI] = 1.474 [1.186–1.831], P<0.001; LODDS3, HR[95%CI] = 2.643 [1.143–6.112], P = 0.023) were independent prognostic factors for OS in patients with Siewert type II AEG (**Table 3**).

**Table 2:** Univariate Cox proportional hazards regression analysis.

Variables	B	SE	Wald	HR	95%CI	*P* value
Age						
≤60	Reference					
>60	0.306	0.055	30.928	1.358	1.219–1.512	<0.001
Sex						
Female	Reference					
Male	0.039	0.067	0.346	1.04	0.912–1.185	0.557
Histology						
Adenocarcinoma	Reference					
SRC	0.182	0.084	4.636	1.199	1.016–1.414	0.031
Others	0.027	0.073	0.135	1.027	0.891–1.184	0.714
Grade						
Grade I	Reference					
Grade II	0.117	0.129	0.825	1.124	0.873–1.448	0.364
Grade III	0.592	0.125	22.404	1.808	1.415–2.310	<0.001
Grade IV	0.465	0.208	5.024	1.592	1.060–2.392	0.025
T						
T1	Reference					
T2	0.698	0.079	78.066	2.01	1.721–2.346	<0.001
T3	0.843	0.088	92.674	2.323	1.957–2.758	<0.001
T4	1.265	0.133	89.8	3.543	2.728–4.603	<0.001
N						
N0	Reference					
N1	0.631	0.062	103.094	1.88	1.665–2.124	<0.001
N2	0.986	0.083	140.15	2.679	2.276–3.154	<0.001
N3	1.525	0.119	162.894	4.593	3.635–5.805	<0.001
M						
M0	Reference					
M1	0.859	0.091	89.859	2.362	1.977–2.821	<0.001
Tumor size						
1~10mm	Reference					
11~30mm	0.414	0.13	10.129	1.513	1.172–1.951	<0.001
31~50mm	0.795	0.129	38.268	2.215	1.722–2.850	<0.001
50mm	0.889	0.129	47.469	2.434	1.890–3.135	<0.001
LNR						
1	Reference					
2	0.674	0.063	114.662	1.962	1.734–2.220	<0.001
3	1.317	0.066	401.921	3.733	3.282–4.246	<0.001
LODDS						
1	Reference					
2	0.67	0.063	113.658	1.955	1.728–2.211	<0.001
3	1.322	0.066	405.217	3.751	3.298–4.266	<0.001
Radiation						
None/Unknown	Reference					
Yes	0.026	0.052	0.247	1.026	0.926–1.137	0.619
Chemotherapy						
None/Unknown	Reference					
Yes	-0.099	0.055	3.28	0.906	0.814–1.008	0.070

**Table 3:** Multivariable Cox proportional hazards regression analysis.

Variables	B	SE	Wald	HR	95%CI	*P* value
Age						
≤60	Reference					
>60	0.447	0.056	64.354	1.563	1.402–1.744	<0.001
Grade						
Grade I	Reference					
Grade II	0.046	0.135	0.118	1.048	0.804–1.365	0.731
Grade III	0.31	0.133	5.457	1.363	1.051–1.767	0.019
Grade IV	0.23	0.213	1.165	1.259	0.829–1.911	0.280
T						
T1	Reference					
T2	0.295	0.091	10.62	1.344	1.125–1.605	<0.001
T3	0.417	0.1	17.559	1.518	1.249–1.845	<0.001
T4	0.64	0.147	18.958	1.897	1.422–2.531	<0.001
**M**	0.505	0.094	29.072			
M0	Reference					
M1	0.505	0.094	29.072	1.658	1.379–1.992	<0.001
Tumor size						
1~10mm	Reference					
11~30mm	0.081	0.135	0.357	1.084	0.832–1.412	0.550
31~50mm	0.196	0.141	1.943	1.216	0.924–1.602	0.163
50mm	0.092	0.143	0.412	1.096	0.828–1.451	0.521
LNR						
1	Reference					
2	0.217	0.113	3.667	1.242	0.995–1.551	0.056
3	0.198	0.426	0.217	1.22	0.529–2.812	0.642
LODDS						
1	Reference					
2	0.388	0.111	12.227	1.474	1.186–1.831	<0.001
3	0.972	0.428	5.167	2.643	1.143–6.112	0.023

### Construction and validation of the nomogram

**Figure 4:**
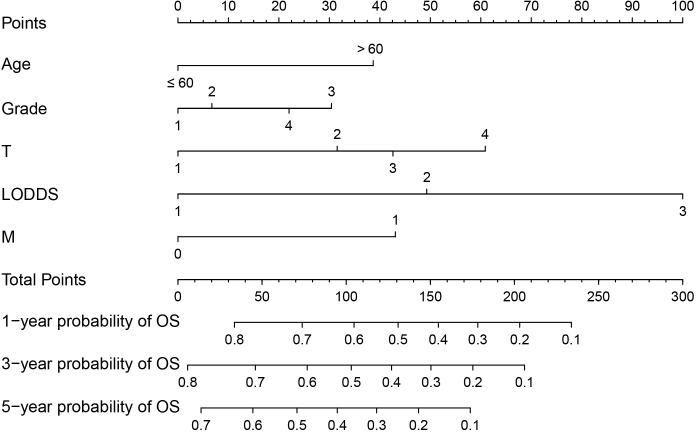
Nomogram predictive model for overall survival (OS) of Siewert type II adenocarcinoma of the esophagogastric junction (AEG) patients. For M, 1 corresponds to M1 stage and 0 corresponds to M0 stage.

**Figure 5:**
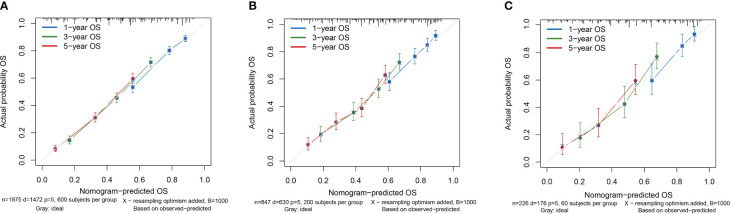
Calibration curves of the nomogram. **(A)** Calibration Curve of the Training Cohort; **(B)** Calibration Curve of the Internal Validation Cohort; **(C)** Calibration Curve of the External Validation Cohort.

**Figure 6:**
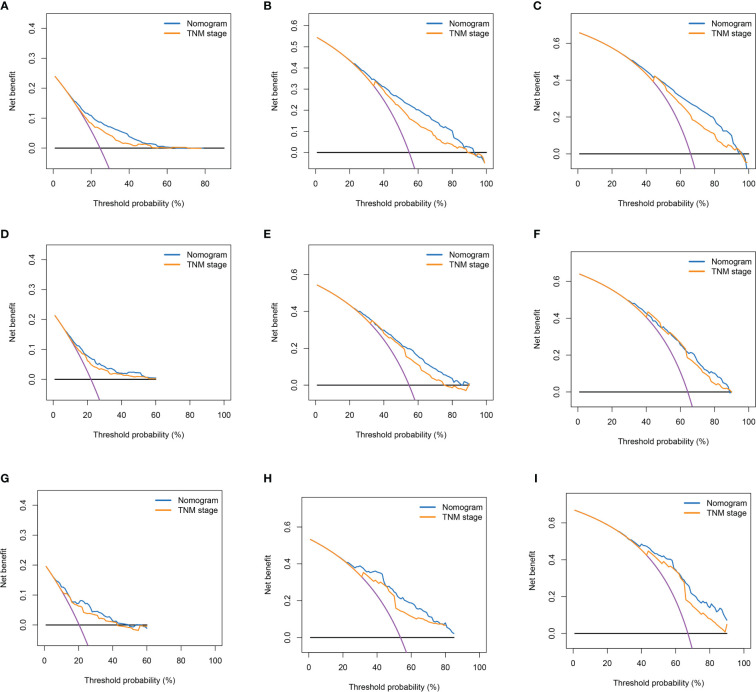
Decision curves of nomogram and TNM stage for 1-year OS, 3-year OS, and 5-year OS. **(A–C)** Decision Curves for 1-year OS, 3-year OS, and 5-year OS in the Training Cohort; **(D–F)** Decision Curves for 1-year OS, 3-year OS, and 5-year OS in the Internal Validation Cohort; **(G–I)** Decision Curves for 1-year OS, 3-year OS, and 5-year OS in the External Validation Cohort.

**Figure 7:**
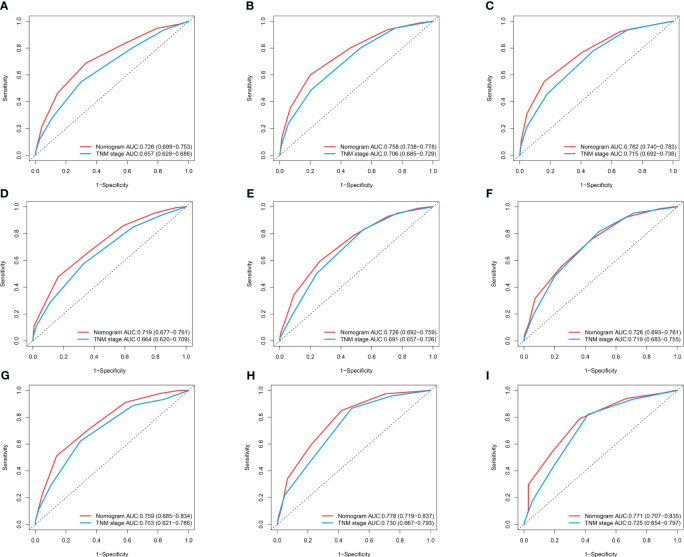
ROC curves of nomogram and TNM stage for 1-year OS, 3-year OS, and 5-year OS. **(A–C)** ROC Curves for 1-year OS, 3-year OS, and 5-year OS in the Training Cohort; **(D–F)** ROC Curves for 1-year OS, 3-year OS, and 5-year OS in the Internal Validation Cohort; **(G–I)** ROC Curves for 1-year OS, 3-year OS, and 5-year OS in the External Validation.

Based on the results of multifactorial Cox regression analysis, the nomogram for predicting OS was established (**Figure 4**), each predictor corresponded to a corresponding score on the scale, and the scores of all the indicators were summed to obtain the total score, which corresponded to the predicted probability of OS of patients at 1, 3, and 5 years, and the larger the total score, the smaller the predicted probability of survival of patients at 1, 3, and 5 years. Calibration plots showed a high degree of agreement between predicted and observed probabilities, indicating significant reliability of the prognostic nomogram (**Figure 5**). Decision curve analysis showed that over a wide range of threshold probabilities, the nomogram consistently provided higher net benefits compared to no assessment (**Figure 6**). The ROC curves showed that the AUC values of the nomogram for the 1-, 3-, and 5-year groups were 0.731, 0.759, and 0.788, respectively, for the training set, 0.725, 0.731, and 0.739 for the internal validation set, and 0.725, 0.731, and 0.739, for the 0.662, 0.769, and 0.788 in the external validation set. In contrast, the AUC values of the TNM staging in the OS prediction model were 0.664, 0.711, and 0.718 in the training set, 0.677, 0.677, and 0.698 in the internal validation set, respectively, and 0.632, 0.731, and 0.739 (**Figure 7**), the nomogram ROC curves showed higher values compared with TNM staging alone, indicating that column-line diagrams have stronger predictive power compared with conventional TNM staging. In addition, we compared the nomogram with the TNM staging system by calculating the NRI versus the IDI. The NRI value of the column-line diagram was 0.494 (95% CI: 0. 368–0.611) in the training cohort, and the corresponding IDI value was 0.08 (95% CI: 0.067–0.093). In the internal validation cohort, the NRI value for the column line graph was 0.339 (95% CI: 0.129 - 0.516) and the corresponding IDI value was 0.0418 (95% CI: 0.025 - 0.059). In the external validation cohort, the NRI value for the column-line graph was 0.575 (95%CI: 0.210- 0.898), with a corresponding IDI value of 0.069 (95%CI: 0.036 - 0.103). Taken together, these metrics confirm that our constructed nomogram accurately predicts OS in Siewert II AEG patients with strong discriminative and predictive performance, and has better predictive ability compared to commonly used TNM staging systems. This highlights its reliability as a valuable tool in clinical practice.

### Risk stratification based on the nomogram

**Figure 8:**
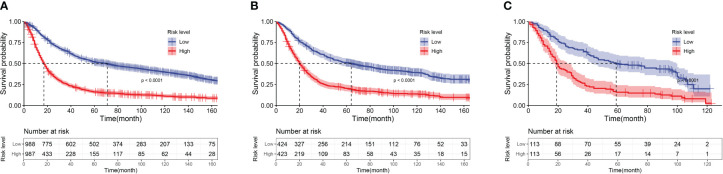
Kaplan-Meier curves of low-risk group and high-risk group patients based on risk score. **(A)** Kaplan-Meier Curves of the Training Cohort; **(B)** Kaplan-Meier Curves of the Internal Validation Cohort; **(C)** Kaplan-Meier Curves of the External Validation Cohort.

According to the nomogram, calculate the risk scores for each patient. Divide patients into low-risk and high-risk groups based on the median as the cutoff point. The average risk score for the low-risk group is 1.691, while the average risk score for the high-risk group is 1.761. Plot the OS survival curves for the training cohort, internal validation cohort, and external validation cohort to assess the performance of our risk model (**Figure 8**). The 5-year OS rate in the training set for the low-risk group is 50.8%, in the internal validation set are 50.4%, and in the external validation cohort are 48.7%. For the high-risk group, the rates are 15.7% in the training set, 19.6% in the internal validation set, and 15% in the external validation cohort. The significant difference in survival rates between the high-risk group (HRG) and low-risk group (LRG) demonstrates the precise accuracy of our risk model.

## Discussion

In recent years, with the increasing trend of gastroesophageal reflux disease (GERD), obesity, smoking and Helicobacter pylori infection, the incidence of AEG has increased significantly. Despite the continuous progress regarding the diagnosis and treatment of AEG, the survival rate of postoperative patients with AEG is still lower than the ideal level, and the overall prognosis is poor. Conventional TNM staging systems contain only a limited number of tumor-related variables, which are limited in specificity and not suitable for personalized analysis. In contrast, the nomogram can take multiple factors into account and has a more accurate predictive ability than traditional TNM staging. SEER database, as one of the world’s recognized tumor databases, provides an effective tool for tumor epidemiology research. So far, a large number of studies have used the SEER database to construct column-line diagrams to predict the prognosis of cancer patients. Xie et al. established a new nomogram to predict the prognosis of lung sarcoma cancer (20). Liu et al. obtained patient data from the SEER database and constructed a nomogram for predicting 1-, 3-, and 5-year OS in patients with invasive micropapillary breast cancer (21). Chen et al. developed and validated a nomogram for predicting 1-, 3-, and 5-year OS and CSS in patients with cervical adenocarcinoma based on data obtained from the SEER database (22). However, few studies have focused on the prognosis of patients with Siewert type II AEG. Therefore, we constructed a nomogram based on the SEER database for predicting OS in Siewert II AEG patients and enhanced the reliability of the model by validating it in an external population.

In this study, we conducted univariate Cox regression analysis for each variable in the training cohort. Since there might be multicollinearity among multiple variables, we included variables with statistical significance (p < 0.05) into the LASSO equation for feature selection. If a group of variables is highly correlated, LASSO regression will select only one variable and reduce other variables to zero, thereby reducing the impact of multicollinearity and decreasing model variance (23, 24). The coefficients for histological type and N stage were zero, so they were excluded from the model. Finally, through multivariable Cox regression analysis of the selected variables, we found that age, tumor grade, T stage, M stage, and LODDS were independent prognostic factors for OS. For validation of the nomogram, we used internal and external validation. The results showed that the nomogram had good predictive performance in both the training set and internal validation set, with high reliability in the external validation set. Compared to the traditional TNM staging, the nomogram had a higher AUC value under the ROC curve, indicating its sensitivity and accuracy in predicting patient prognosis. Furthermore, through calculation of the NRI and IDI, we compared the nomogram with the TNM staging system. The results showed that our constructed nomogram had better predictive ability compared to the commonly used TNM staging system. Finally, we divided patients into high-risk and low-risk groups based on the risk scores from the nomogram and observed significant differences in survival curves among different validation cohorts, validating the effectiveness of the nomogram in risk stratification.

LODDS is a new index to assess lymph node status by considering the number of lymph node metastases and positive. The ratio of lymph nodes to negative lymph nodes provides more accurate cancer staging. In contrast, traditional N staging usually simply counts the number of positive lymph nodes, and several studies have shown that LODDS is superior to N staging alone in identifying lymph node metastasis and providing an accurate prognosis for cancer patients. Dimitrios Prassas et al. found that LODDS staging was superior to N staging in terms of prognostic performance and ability to identify patients with pancreatic cancer (25). Gu et al. found that the LODDS staging system was superior to other lymph node classifications in predicting the prognosis of patients undergoing radical gastric cancer surgery in a multi-institutional analysis of 7620 patients in China (26); Tang et al. showed that the LODDS was superior to N-staging and PLN in prognostic efficiency as an independent prognostic factor for medullary thyroid carcinoma and that the LODDS-based columnar line graphs had better performance than the AJCC TNM staging system in predicting cancer-specific survival after surgery for medullary thyroid carcinoma (27). Li et al. compared the predictive efficiency of LNM indexes by constructing a multifactorial Cox regression model, and found that LODDS was superior to N classification, PLN and LNR in prognosticating OS and CSS in prostate cancer patients (28); Zhao et al. compared the effectiveness of N classification, PLN, LNR and LODDS in predicting cancer-specific survival in patients with small cell lung cancer and found that the LODDS model showed the highest accuracy in predicting cancer-specific survival in patients with small cell lung cancer compared to N classification, PLN and LNR (29).

Study shows advanced age is a risk factor for poor prognosis in cancer patients (30). In addition, elderly patients are usually accompanied by various health problems, such as malnutrition, cardiovascular and cerebrovascular diseases, diabetes mellitus, hypertension, etc., and have lower treatment tolerance, which may prompt physicians to choose more conservative treatments or shorten the course of treatment, affecting the outcome of the therapy (31). In addition, older patients may be more prone to surgical and treatment-related complications, leading to a poorer prognosis (32). In a cohort study, Cao et al. found that older AEG patients had a worse prognosis than younger patient (33); Poorly differentiated tumors lose the morphology and function of normal cells and are usually more aggressive, with a higher likelihood of recurrence and distant metastasis. In a study by Shi et al. tumor grade was found to be an independent risk factor for overall and cancer-specific survival in patients with gastric cardia cancer (34); Wang et al. observed through their study that poor tumor differentiation resulted in a worse prognosis for patients with adenocarcinoma of the gastric cardia (35).

T-staging is used to describe the depth of tumor infiltration and the degree of invasion of surrounding tissues or organs. Tumors with a high degree of invasion are more difficult to remove completely and therefore require more complex surgical procedures and treatments such as radiotherapy, chemotherapy, or targeted therapies, and this increase in therapeutic difficulty often leads to greater risk and a longer course of treatment, which may have an impact on the patient’s physical functioning (36). It has also been shown that the depth of tumor infiltration is closely related to tumor recurrence (37). In addition, a higher T stage leads to an increased risk of distant metastasis, and once the tumor develops distant metastasis, it can cause a compressive effect and an imbalance of physiological homeostasis, which often leads to a poor outcome (38, 39).

But here, we also need to explain that although there are fewer studies of the same type, some researchers have conducted similar explorations. Zhangjian Zhou (40) et al. also used the SEER database to establish a nomogram, but the evaluation indicators of their nomogram were too complex and were not conducive to actual clinical use. In addition, we note that their study did not conduct a comprehensive evaluation of the performance of the nomogram, which is a shortcoming of their study. The research by Jun Wang (41) et al. is generally more similar to ours, but in comparison, our research has a larger sample size, which can better enhance the objectivity of our conclusions, and our model, regardless of the performance of the internal validation set and the external validation set is not only better than the traditional Stage staging, but also better than their team’s research. All the above are the strengths of this study.

The reason we used data from 2012 to 2017 to externally validate our model is that by using a newer cohort of patients from another independent data source, we were able to test the performance of our prognostic model in more recent cases and verify its stability and relevance as diagnostic and therapeutic paradigms evolve. There were no statistically significant differences between the two cohorts in key clinical indicators such as demographic characteristics, tumor staging, and treatment modalities. This consistency suggests that despite advances in medical technology and changes in clinical guidelines, the core features of patients and their treatments remain sufficiently similar to ensure effective comparisons and reliable conclusions. Therefore, we believe that using data from 2012 to 2017 for external validation of our model is both appropriate and indicative of its applicability to current clinical practice. This validation strategy enhances the generalizability of our study findings and supports the utility of the model in the ongoing clinical setting.

Overall, this study constructed a novel nomogram based on LODDS to predict the OS of Siewert II AEG patients and provided an in-depth and comprehensive analysis of the prognosis of Siewert II AEG patients, which provides strong support for clinical practice, provides a more accurate basis for individualized treatment of patients, and contributes to the development of a more effective treatment plan. However, it should be noted that there are still some limitations in this study, first of all, the SEER database lacks patients’ clinical information, perioperative blood markers and imaging characteristics. These are important for prognostic assessment of malignant tumors. In addition, our study was retrospective; therefore, a prospective study is necessary to further validate our findings and confirm the validity of nomogram in patients with Siewert type II AEG.

## Conclusion

We utilized clinical data from the SEER database to identify factors associated with survival in patients with Siewert type II AEG. Subsequently, a new nomogram based on the LODDS was developed that accurately predicts OS in patients with Siewert type II AEG. Our findings suggest that nomogram outperform TNM staging in terms of predictive power, providing clinicians with a simple yet powerful tool that can be used to assess patients’ prognostic risk at a more granular level and to develop personalized treatment plans.

## Data availability statement

Publicly available datasets were analyzed in this study. This data can be found here: https://seer.cancer.gov/data-software/.

## Ethics statement

The studies involving humans were approved by The institutional review board of the ethics committee ofDandong central hospital (No. DDSZXYY-2024-27). The studies were conducted in accordance with the local legislation and institutional requirements. Written informed consent for participation was not required from the participants or the participants' legal guardians/next of kin in accordance with the national legislation and institutional requirements.

## Author contributions

XY: Conceptualization, Investigation, Software, Writing – original draft, Data curation, Methodology. CB: Conceptualization, Investigation, Software, Writing – original draft, Data curation, Methodology. YY: Data curation, Investigation, Writing – original draft. XG: Investigation, Methodology, Writing – original draft. KW: Investigation, Supervision, Writing – original draft. HY: Data curation, Investigation, Validation, Writing – original draft. XL: Supervision, Validation, Writing – original draft, Writing – review & editing.
